# TCF/β-catenin plays an important role in *HCCR-1 *oncogene expression

**DOI:** 10.1186/1471-2199-10-42

**Published:** 2009-05-12

**Authors:** Goang-Won Cho, Mi-Hwa Kim, Seung Hyun Kim, Seon-Ah Ha, Hyun Kee Kim, Sanghee Kim, Jin W Kim

**Affiliations:** 1 Department of Neurology, College of Medicine, Hanyang University, 17 Haengdang-dong, Seongdong-gu, Seoul, 133-791, Korea; 2 Molecular Genetic Laboratory, College of Medicine, The Catholic University of Korea, Seoul, 137-040, Korea; 3 Department of Obstetrics and Gynecology, College of Medicine, The Catholic University of Korea, Seoul, 137-040, Korea

## Abstract

**Background:**

Oncogene *HCCR-1 *functions as a negative regulator of the p53 and contributes to tumorigenesis of various human tissues. However, it is unknown how *HCCR-1 *contributes to the cellular and biochemical mechanisms of human tumorigenesis.

**Results:**

In this study, we showed how the expression of *HCCR-1 *is modulated. The luciferase activity assay indicated that the *HCCR-1 *5'-flanking region at positions -166 to +30 plays an important role in *HCCR-1 *promoter activity. Computational analysis of this region identified two consensus sequences for the T-cell factor (TCF) located at -26 to -4 (Tcf1) and -136 to -114 (Tcf2). Mutation at the Tcf1 site led to a dramatic decrease in promoter activity. Mobility shift assays (EMSA) revealed that nuclear proteins bind to the Tcf1 site, but not to the Tcf2 site. LiCl, Wnt signal activator by GSK-3β inhibition, significantly increased reporter activities in wild-type Tcf1-containing constructs, but were without effect in mutant Tcf1-containing constructs in HEK/293 cells. In addition, endogenous *HCCR-1 *expression was also increased by treatment with GSK-3β inhibitor, LiCl or AR-A014418 in HEK/293 and K562 cells. Finally, we also observed that the transcription factor, TCF, and its cofactor, β-catenin, bound to the Tcf1 site.

**Conclusion:**

These findings suggest that the Tcf1 site on the *HCCR-1 *promoter is a major element regulating *HCCR-1 *expression and abnormal stimulation of this site may induce various human cancers.

## Background

Proto-oncogenes normally help regulate cell growth and differentiation under well-controlled conditions, including mitogenic signal transductions in cells [1,2]. Uncontrolled expression of proto-oncogenes due to mutations or activation of signaling can give rise to a tumor-inducing agent, which is known as an oncogene [2,3]. For more than a decade, there has been a focus on the transcriptional regulation of oncogenes or proto-oncogenes in search of therapeutic clues against cancers which are induced by over-transcription of their oncogenes.

Wnt is known as a proto-oncogene and its signaling pathway is a complex network of proteins with roles in embryogenesis [4-6] and cancer [7]. Wnt and its signaling pathway is also involved in normal physiologic processes, including cell polarity [8], axon guidance [9], and stem cell growth factor [10]. Two Wnt pathways have been identified, the canonical and non-canonical pathways. More than 90% of colorectal cancers and other digestive cancers are associated with defects in the canonical Wnt signaling pathway by mutations in APC [11,12], AXIN1 [13], or CTNNB1 [7]. These mutations make it impossible to assist GSK-3β in phosphorylation and in rapid degradation of β-catenin through the ubiquitin pathway as a result of accumulation of β-catenin in the cytoplasm and formation of a complex with TCF in the nucleus, which initiates transcription of the Wnt target genes [14,15].

The human cervical cancer oncogene 1 (*HCCR-1*) has been identified as a novel oncogene with strong tumorigenic features in nude mice [16]. *HCCR-1 *is post-translationally localized in the mitochondria, sub-compartmentally in its outer membrane [17,18], and may functionally regulate the p53 tumor-suppressor gene negatively [16,19]. *HCCR-1 *is also overexpressed in various types of human malignancies, including colorectal cancer [18]. However, it is not known how *HCCR-1 *expression is modulated. In this study, we characterized the proximal promoter region of *HCCR-1 *to elucidate the mechanism of expression of the oncoprotein, HCCR-1.

## Results and Discussion

### Characterization of the human HCCR-1 5'-flanking sequences

Previous work has identified the initiation site for transcription and the promoter region of the *HCCR-1 *gene [20]. Computational analysis has shown that the HCCR-1 promoter contains a TATA box, a CAAT box, and the putative DNA binding sites for various transcriptional factors [20].

**Figure 1:**
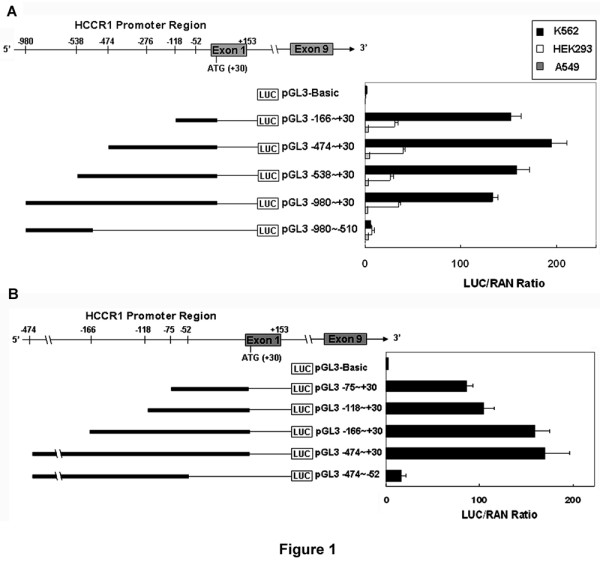
**Expression of the truncated *HCCR-1 *promoter in K562, HEK/293, or A549 cells**. The DNA constructs containing various lengths of the *HCCR-1 *promoter region were cloned into upstream of the firefly luciferase (LUC) reporter gene. Each luciferase reporter construct shown in the diagram was co-transfected in K562 (*A *and *B*; filled bars), HEK293 (*A*; open bars), and A549 (*A*; shadow bars) cells with the pRL-CMV normalizing reporter plasmid encoding with the *renilla *(REN) gene. The horizontal axis shows the ratio of luciferase to *renilla *activity normalized by LUC/REN (= 100%) displayed by cells co-transfected with pRL-CMV and pGL3-control. We designated each recombinant vector as pGL3X~Y, where X is the first base and Y the last base of each truncated promoter (*n *> 5 per construct).

To characterize the *HCCR-1 *promoter, fragments from positions -980, -538, -474, and -166 to position +30 (end of the 5'UTR region) and position -980 to position -510 were cloned into upstream of a luciferase reporter gene and assayed for their transcriptional activities in either chronic myelogenous leukemia K562, HEK/293, or lung cancer A549 cells (Figure 1A). The activities from all the constructs tested were high in the K562, but weak in HEK/293 and nearly undetectable in A549 cells, indicating that the activity of the *HCCR-1 *promoter is constitutively enhanced in K562 cells. Northern blot analysis showed that *HCCR-1 *expression is high in K562 and weak in A549 cells, which is consistent with the present work [16]. Transient transfection of a reporter fragment containing -474 to +30 of human *HCCR-1 *(referred to hereafter as 'pGL3-474~+30') had 97.5 times higher promoter activity than the reporter gene alone (pGL3-Basic) in K562 cells (Figure 1A). However, the shortest fragment (pGL-166~+30) had high promoter activity, while the deleted mutant (pGL-980~-510) had very weak activity (Figure 1A), suggesting that the *HCCR-1 *promoter region from -166 to +30 plays an important role in *HCCR-1 *gene expression.

To further characterize the *HCCR-1 *promoter region from -166 to 30, promoter fragments from positions -118 and -75 to position +30 and from positions -474 to position -52 were cloned into upstream of a luciferase reporter gene and assayed for their transcriptional activity in K562 cells (Figure 1B). The fragment (pGL-166~+30) had 1.85 times higher promoter activity than pGL-75~+30 and 1.53 times higher promoter activity than pGL3-118~+30 in K562 cells. However, this activity was dramatically reduced by the deleted mutant (pGL-474~-52) which was removed at positions -52 to +30 (Figure 1B). These results suggest that the *HCCR-1 *5'-flanking region at positions -52 to +30 is required for *HCCR-1 *promoter activity and this region may possess important elements enhancing *HCCR-1 *transcription.

### The Tcf1 Site is involved in the activation of the HCCR-1 promoter

**Figure 2:**
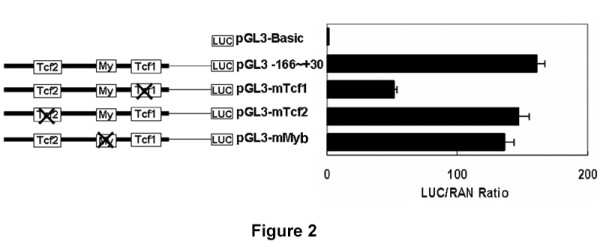
**Mutational analysis of the *HCCR-1 *promoter**. The diagrams depict locations of the predicted transcriptional elements in the *HCCR-1 *promoter. When mutated, the diagrams are indicated by a cross. "Tcf" and "My" indicate the consensus sequences for the TCF and C-Myb elements, respectively. Each luciferase reporter construct was co-transfected in K562 cells with pRL-CMV. The horizontal axis shows the ratio of luciferase (LUC) to *renilla *(REN) activity normalized by LUC/REN (= 100%) displayed by cells co-transfected with pRL-CMV and pGL3-control (*n *> 4 per construct).

The MatInspector [21] identified two T-cell factor (TCF) binding sites, designated as Tcf1 and Tcf2 in Figure 2[15,22,23] and one c-myb site designated as My in Figure 2[24,25] within the -166 to +30 region of the *HCCR-1 *promoter. To investigate the role of these elements in the modulation of transcriptional activity, each was inactivated by site-directed mutagenesis and cloned into upstream of a luciferase reporter gene. Each mutated construct (pGL3-mTcf1, -mTcf2, and -mMyb) was transiently expressed in K562 cells and the luciferase activity was measured. Mutation of the Tcf2 or c-myb-binding sites did not significantly modify luciferase expression (Figure 2). In contrast, mutation of the Tcf1 site significantly affected *HCCR-1 *promoter activity. We thus focused on the Tcf1 site for further investigations.

### Nuclear proteins extracted from K562 cells recognize the sequences of the Tcf1 site

**Figure 3:**
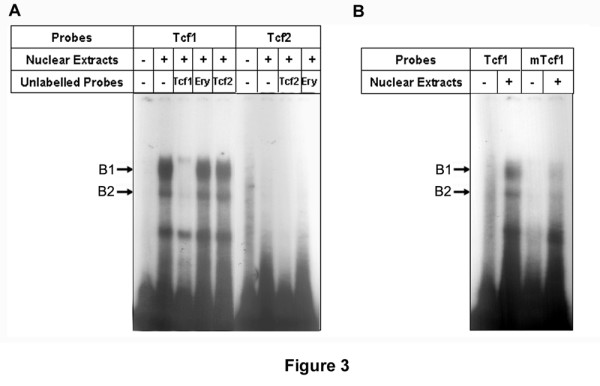
**DNA-binding activity at the Tcf1 site**. EMSA were performed using K562 nuclear extracts and dsDNA harboring either a consensus site for HCCR-1 wild-type Tcf1, Tcf2 element (*A*), and a mutated sequence mTcf1 (*B*). To show the specificity of the bands, 10 times molar excess of cold double-stranded oligonucleotide carrying the consensus Tcf1 and Tcf2 sites was used for competition or Ery for non-competition. B1 indicates the DNA-protein complex with probe Tcf1 and B2 indicates less specific DNA-protein complex. The presence or absence of a component in the assay is indicated by a "+" or "-", respectively.

To confirm that Tcf1 plays a role in *HCCR-1 *promoter activation, we then performed an electrophoretic mobility shift assay (EMSA) in the K562 nuclear extracts by using double-stranded oligonucleotides harboring either the *HCCR-1 *wild-type sequences, designated as Tcf1 (-26~-4), Tcf2 (-136~-114), or a mutated Tcf1 sequence (mTcf1). Two retarded bands were obtained with the radiolabeled probe carried the wild-type Tcf1 site, but no band in the labeled probe carrying the Tcf2 site (Figure 3A). The band was abolished by pre-incubation with a 10 times molar excess of cold Tcf1 probe, but not abolished with cold probes that carried Tcf2 or Ery (erythroid krueppel like factor; -294~-272) probes, which were used as a non-competitor (Figure 3A). The band also disappeared when using the probe encoding the mutated Tcf1 site, mTcf1 (Figure 3B), suggesting that proteins included in the K562 nuclear extracts recognized and bound to the wild-type specific sequences which are situated in the Tcf1 site on the *HCCR-1 *promoter.

As shown in Figure 1B, the reporter fragment containing -166 to +30 of human *HCCR-1 *was significantly increased in comparison with the fragment containing -118 to +30. However, EMSA assays revealed no major band when the radiolabeled probe carried the *HCCR-1 *wild-type Tcf2 sequences (Figure 3A), suggesting that there is another element downstream from the Tcf2 region to -166 of the *HCCR-1 *promoter region. We have searched this region, and detected three shifted bands by the EMSA method (unpublished data). These bands disappeared in the presence of a cold competitor and were not with the non-competitor. Site-directed mutagenesis work also revealed that the region is important for *HCCR-1 *expression, but further study is required to gain better insight into the biologic significance in *HCCR-1 *expression.

### HCCR-1 promoter was activated by LiCl, an inhibitor of GSK-3β

**Figure 4:**
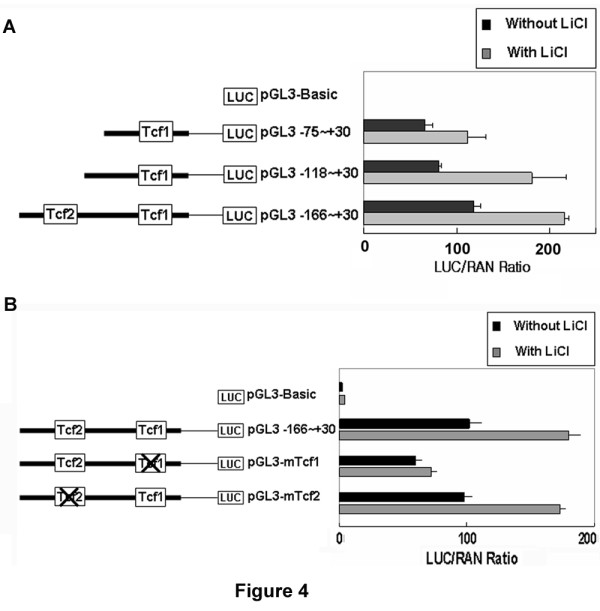
**TheTcf1 site on the *HCCR-1 *promoter was sensitive to the GSK-3β inhibitor, LiCl**. The truncated constructs (*A*) or mutated constructs (*B*) of the *HCCR-1 *promoter region were transfected into K562 cells with pRL-CMV as an internal control. Cells were then treated with or without 5 mM LiCl for 24 hours and subjected to dual luciferase analysis (*n *> 4 per construct).

Lithium has been shown to stabilize β-catenin via inhibition of GSK-3β [14,26], and as a result, activates TCF. To examine whether TCF activation by treatment with 5 mM LiCl stimulated *HCCR-1 *promoter expression [26], we performed reporter assays with deletion constructs containing Tcf1 or both Tcf1 and Tcf2 sites in K562 cells (Figure 4A). LiCl treatment increased reporter activities 1.7- to 2.25-fold with constructs pGL3-75~+30,-118~+30 which contain the Tcf1 site, and 1.8-fold with pGL3-166~+30 containing the Tcf1 and Tcf2 sites (Figure 4A).

Another reporter assay with constructs pGL3-mTcf1 or -mTcf2, which are mutated at the Tcf1 or Tcf2 sites within the -166 to +30 region, was performed to verify Tcf1 involvment in *HCCR-1 *promoter activity. pGL3-mTcf1 markedly attenuated the induction of *HCCR-1 *promoter activity by treatment with LiCl in comparison with wild-type pGL3-166~+30, but there was no significant change at the mutation of the Tcf2 site (pGL3-mTcf2; Figure 4B). These results suggest that Tcf1 appears to be important for LiCl-induced activation of the *HCCR-1 *promoter.

### Endogenous HCCR-1 transcripts were increased by GSK-3β inhibitors

**Figure 5:**
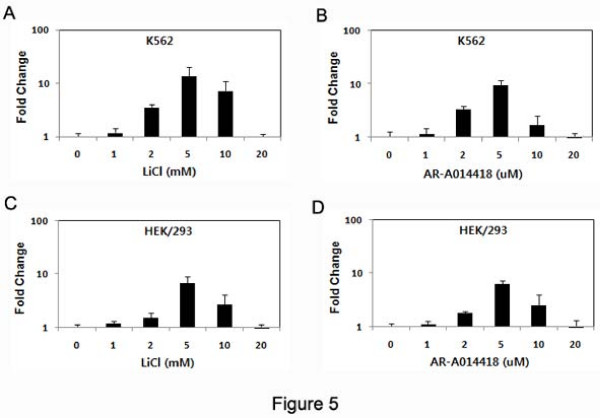
**Endogenous expression of *HCCR-1 *on activated Wnt/β-catenin signal by treatment with the GSK-3β inhibitors, LiCl or AR-A014418**. Total RNAs were prepared from K562 (*A and B*) and HEK/293 (*C and D*) cells which were treated with various concentration of LiCl (*A and C*) or AR-A014418 (*B and D*) for 24 hours in respectively. Five μg of total RNAs were used for cDNA synthesis and subjected to real-time PCR analysis with human specific primers for HCCR-1 and β-actin (mean ± S.D., *n *= 3).

To verify activation induced by stimulation of the Wnt/β-catenin signal on endogenous *HCCR-1 *mRNA expression, K562 or HEK293 cells were treated with various concentration of LiCl (0, 1, 2, 5, 10, and 20 mM) [26] or AR-A014418 (0, 1, 2, 5, 10, and 20 μM) [27] for 24 h and the levels of expression were analyzed by real-time PCR. As shown in Figure 5A, the stimulated K562 cells (2, 5 or 10 mM) were increased the expression of *HCCR-1 *compared with unstimulated cells (0 mM), and the expression was increased 13.7-fold in the cells treated with 5 mM LiCl and 9.3-fold with 5 μM AR-A014418 (Figure 5A and 5B). The expression of *HCCR-1 *mRNA was increased 6.6-fold in the HEK/293 cells treated with 5 mM LiCl and 6.26-fold in same cells with 5 μM AR-A014418 (Figure 5C and 5D). These results suggest that the expression of *HCCR-1 *is modulated by the activation of transcription factor, TCF, induced by β-catenin stability.

### TCF and β-catenin are bound to the Tcf1 site on the HCCR-1 promoter

**Figure 6:**
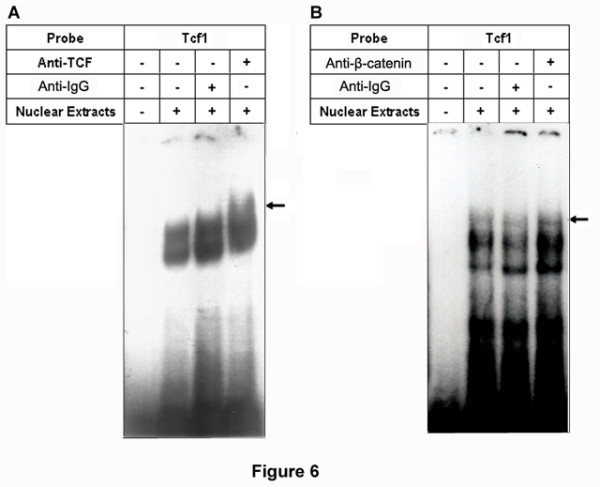
**TCF and β-catenin bound with the Tcf1 element on the *HCCR-1 *promoter region**. Super-shift assays using K562 nuclear lysates were tested with labeled oligo probes Tcf1 and 0.4 μg antibodies against either TCF (*A*) or β-catenin (*B*). Anti-IgG indicate non-specific antibody for control. Arrows indicate super-shifted bands.

We have shown that the Tcf1 site on the *HCCR-1 *proximal 5'-flanking region regulates endogenous *HCCR-1 *expression. We next investigated whether TCF and its cofactor (β-catenin) could physically bind with the *HCCR-1 *promoter using a super-shift assay with the radiolabeled oligo Tcf1 probes and nuclear extracts from K562. As shown in Figure 6, incubation with either anti-TCF (Figure 6A) or anti-β-catenin (Figure 6B) antibodies resulted in a super shift of the DNA-protein complexes, whereas incubation with a nonspecific IgG did not shift the DNA-protein complexes, confirming that TCF and β-catenin are bound to the Tcf1 site on the *HCCR-1 *promoter.

Wnt signaling is constitutively active in the majority of human colorectal cancers by mutations which lead to increased β-catenin protein expression [7,11-13], resulting in nuclear accumulation of β-catenin, which subsequently complexes with TCF transcription factors and activation of downstream β-catenin/TCF target genes, including Bcl-2 family genes [28]. HCCR-1 is also overexpressed in colorectal cancer and interacts with DP1 on the mitochondrial membrane [18]. Overexpressed *HCCR-1 *may contribute to the cellular and biochemical mechanisms of human tumorigenesis [18]. In this study, we characterized the *HCCR-1 *promoter and elucidated the mechanism of expression of the strong oncoprotein *HCCR-1*. We found that *HCCR-1 *expression is directly modulated by TCF/β-catenin signaling and may play a role in human cancer.

## Conclusion

*HCCR-1 *has been isolated as an oncogene candidate and is overexpressed in various cancers [16,18,19,29]. To understand the modulation of *HCCR-1 *expression, several deleted mutants of the proximal promoter region of *HCCR-1 *were constructed. Reporter gene assays in K562 cells demonstrated that the proximal promoter region at nucleotides -166 to +30 have a pivotal role in *HCCR-1 *transcription. Mutagenesis of the Tcf1 site significantly decreased the reporter activity compared with its wild-type. Our data also indicate that the transcription of *HCCR-1 *is activated by treatment with LiCl, the reagent sensitive to GSK-3β. Site-directed mutagenesis on the Tcf1 site makes the *HCCR-1 *promoter activity insensitive to the stimulation of β-catenin/TCF signaling by LiCl. In addition, stimulation with LiCl or AR-A014418 also increased endogenous *HCCR-1 *expression in K562 and HEK/293 cells, suggesting that the Tcf1 site on the *HCCR-1 *proximal promoter is a major element for *HCCR-1 *expression. Finally, we also observed that the transcription factor, TCF, and its cofactor (β-catenin) are indeed bound to the Tcf1 site.

## Methods

### Cell culture

Human embryonic kidney (HEK) 293 (ATCC CRL-1573), K562 chronic myelogenous leukemia (ATCC CCL-243), and A549 lung cancer (ATCC CCL-185) cells were obtained from the American Type Culture Collection (Manassas, VA, USA). The HEK/293 cells were maintained at 37°C and 5% CO_2 _in DMEM (Gibco, Grand Island, NY, USA) supplemented with 10% FBS and 1% penstrep (Gibco). K562 and A549 were maintained at 37°C in 5% CO_2 _in RPMI (Gibco) containing 10% FBS and 1% penstrep.

### Isolation of the promoter region and DNA transfection

Eight DNA fragments from the *HCCR-1 *promoter region (Figures 1A and 1B) were synthesized using a *Pfu *DNA polymerase (MBI Fermentas, MD). The lambda phage DNA containing the *HCCR-1 *genomic DNA was amplified using the primers G1-R and G1-F for pGL3-75~+30, G1-R and G2-F for pGL3-118~+30, G1-R and G3-F for pGL3-166~+30, G1-R and G4-F for pGL3-474~+30, G2-R and G4-F for pGL3-474~-52, G1-R and G5-F for pGL3-538~+30, G1-R and G6-F for pGL3-980~+30, and the primers G3-R and G6-F for pGL3-980~-510 (Additional file 1: Table S1). The primer sequences used in these PCR reactions are shown in Additional file 1: Table S1 (GenoTech, Daejeon, Korea). The PCR cycling parameters were as follows: initial denaturation at 94°C for 2 min; 30 cycles of 30 s at 94°C for denaturation; 30 s at 55°C for primer annealing; 1 min at 72°C for extension; and final extension at 72°C for 10 min. The PCR products were subcloned into the pGL3-Basic vector (Promega, Madison, WI, USA) at the *Kpn*I and *Xho*I sites and confirmed by sequencing (GenoTech). All plasmids and recombinant plasmids were prepared using Qiagen columns (Qiagen Inc, Valencia, CA, USA).

For DNA transfection, K562, HEK/293, and A549 cells were co-transfected with 200 ng pGL3-basic vector containing the *HCCR-1 *promoter regions and 40 ng pCMV-RL, as an internal standard, using 3 μl Lipofectamine 2000 (Invitrogen, Carlsbad, CA, USA), according to the manufacturer's protocol.

### Luciferase (LUC) reporter assay

K562, HEK/293, and A549 cells were co-transfected with the pGL3-basic vector containing various *HCCR-1 *promoter regions and pCMV-RL. To investigate the effect of the Wnt-signal pathway on HCCR-1 expression, cells were treated with 5 mM LiCl (Sigma-Aldrich Corporation, St. Louis, MO, USA) for 24 h, then harvested. After lysis, the cell suspensions were centrifuged at 12,000 × g. Firefly and *Renilla *luciferase activities of cell lysates were measured according to the manufacturer's instructions for the Dual Luciferase Assay System (Promega) in a Turner TD-20/20 luminometer (Turner Designs, Sunnyvale, CA, USA). The relative firefly luciferase activity was calculated by normalizing transfection efficiency to *Renilla *luciferase activity for each cell type. An error bar is used to show the SD derived from more than four independent experiments.

### Mutagenesis

Pairs of complimentary oligonucleotides containing the desired mutations were synthesized (GenoTech). Mutated plasmids were obtained using the QuikChange site-directed mutagenesis kit (Stratagene, La Jolla, CA, USA). The mutagenesis reaction was performed following the manufacturer's protocol using the pGL3-166~+30 plasmid for pGL3-mTcf1, pGL3-mTcf2, and pGL3-mMyb as a template. Mutagenesis products were obtained as follows: pGL3-mTcf1 using primers mT1-F and mT1-R; pGL3-mTcf2 using mT2-F and mT2-R; and pGL3-mMyb using mMyb-F and mMyb-R (Additional file 1: Table S2). The nucleotide sequence of each construct was verified by automated sequencing (GenoTech). All primer sequences used in mutagenesis are shown in Additional file 1: Table S2.

### Nuclear protein extraction

Nuclear extracts were prepared from K562 cells according to the manufacturer's instructions using a nuclear extraction kit (Sigma-Aldrich Corporation), with minor modifications. Briefly, 1 × 10^7 ^cells were washed twice with ice-cold phosphate-buffered saline and resuspended in 500 μl hypotonic lysis buffer (10 mM HEPES [pH 7.9], 1.5 mM MgCl_2_, 10 mM KCl, 1 mM dithiothreitol, and protease inhibitor cocktail) for K562 and left on ice for 15 min. Cells were centrifuged for 5 min at 420 × g and resuspended again in 200 μl of the hypotonic lysis buffer. After the lysates were passed 5 times through a 27-gauge needle, nuclei were recovered by centrifugation at 10,000 × g for 20 min at 4°C. The nuclei were resuspended in 80 μl of extraction buffer (60 mM HEPES [pH 7.9], 1.5 mM MgCl_2_, 420 mM NaCl, 0.2 mM EDTA, 25% glycerol, 1 mM dithiothreitol, and protease inhibitor cocktail) and incubated on ice for 30 min. The mixture was centrifuged at 20,000 × g at 4°C for 5 min, and the supernatant (the nuclear extract) was collected and stored at -70°C until use. Protein concentrations of the nuclear extract were determined by the Bradford assay.

### Electrophoretic mobility shift assay (EMSA)

EMSA was performed using 3.0 μg of nuclear extracts prepared from K562 cell lines. Double-stranded oligonucleotide probes (Tcf1 for primers emT1-F and emT1-R, Tcf2 for emT2-F and emT2-R, Ery for emEry-F and emEry-R, and mTcf1 for mT1-F and mT1-R; Additional file 1: Table S2) were generated by annealing an antisense strand to its complementary matched sense strand and labeled with [γ-^32^P]ATP (3,000 Ci/mmol [10 mCi/ml]; NEN Life Science products) and T4 polynucleotide kinase (Takara). The Gel Shift Assay System kit (Promega) was used for the binding reaction, which was performed as recommended by the manufacturer. The ^32^P-labeled probes were added to the nuclear extract and reacted for 20 min.

For super-shift analysis, 0.4 μg of a nonspecific goat anti-rabbit IgG, a rabbit anti-human TCF polyclonal antibody or a rabbit anti-human β-catenin polyclonal antibody (Santa Cruz Biotechnology, Santa Cruz, CA, USA) was added to the nuclear extract mixture containing ^32^P-labeled probes and reacted for 20 min. The reaction mixtures were resolved on a 6% non-denaturing polyacrylamide gel in 0.5 times Tris borate-EDTA buffer at 250 V for 30 min. The gel was dried and exposed to an x-ray film (Kodak) at -70°C for ~6 h. After that, the bands were visualized by autoradiography. The putative consensus oligonucleotides and mutant oligonucleotides are listed in Additional file 1: Table S2.

### Real-time PCR and Reverse Transcription-Polymerase Chain Reaction (RT-PCR)

K562 and HEK/293 cells were incubated with serum-free media for 24 h and then treated with LiCl (0, 1, 2, 5, 10, or 20 mM; Sigma-Aldrich Corporation) [26] or AR-A014418 (0, 1, 2, 5, 10, or 20 μM; Sigma-Aldrich Corporation) [27] for 24 h and harvested. Total RNAs were prepared using a TRIZOL Reagent (Invitrogen) according to the manufacturer's recommendation. 3~5 μg of total RNA were reverse-transcribed using RevertAid™ M-MuLV reverse transcriptase (MBI Fermentas, USA), 0.2 μg random primer (Invitrogen, USA), 1 mM *d*NTPs, and the supplied buffer. Each group of the first strand cDNAs was amplified using Power SYBR Green PCR master mix (Abioscience, UK) with the primer pairs RT-F and RT-R for the HCCR-1 gene or Act-F and Act-R for β-actin (Additional file 1: Table S1). The real-time PCR cycling parameters were as follows: initial denaturation at 95°C for 10 min; 40 cycles of 15 sec at 95°C for denaturation, 1 min at 60°C for primer annealing and extension. An error bar is used to show the S.D. derived from three independent experiments.

## Abbreviations

HCCR-1: human cervical cancer oncogene 1; TCF: T-cell factor; EMSA: electrophoretic mobility shift assay; GSK-3β: Glycogen synthase kinase-3beta; K562: chronic myelogenous leukemia 562; HEK/293: human embryonic kidney 293; DMEM: Dulbecco's modified Eagle's medium; LUC: luciferase; RAN: *renilla*; UTR: untranslated region; DP1: deleted in polyposis 1.

## Authors' contributions

JWK and SHK designed the studies, analyzed data and wrote the manuscript. GC, MK, SH, HKK and SK performed the luciferase activity assay, computational analysis, mutation experiment, and EMSA assay. All authors contributed to editing the manuscript.

## Supplementary Material

Additional file 1 **Tables**. Table S1 and Table S2. Click here for file
