# Supplementary material for: TCF/β-catenin plays an important role in HCCR-1 oncogene expression
Source: BMC Mol Biol. 2009 May 12;10:42. doi: 10.1186/1471-2199-10-42 (PMC2693525; doi:10.1186/1471-2199-10-42)
Supplement: Additional file 1 — Tables. Table S1 and Table S2. [file 1471-2199-10-42-S1.doc]

Tables

**Table S1:** Oligonucleotides for promoter cloning and RT-PCR


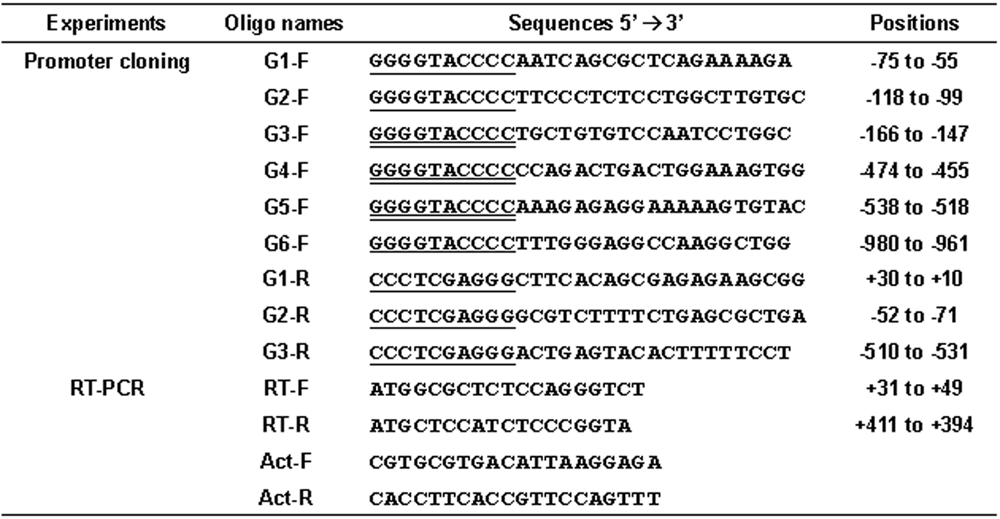


The single or double underlines denote the *Xho*I or *Kpn*I restriction enzyme sites, respectively.

**Table S2:** Oligonucleotides for EMSA and mutagenesis


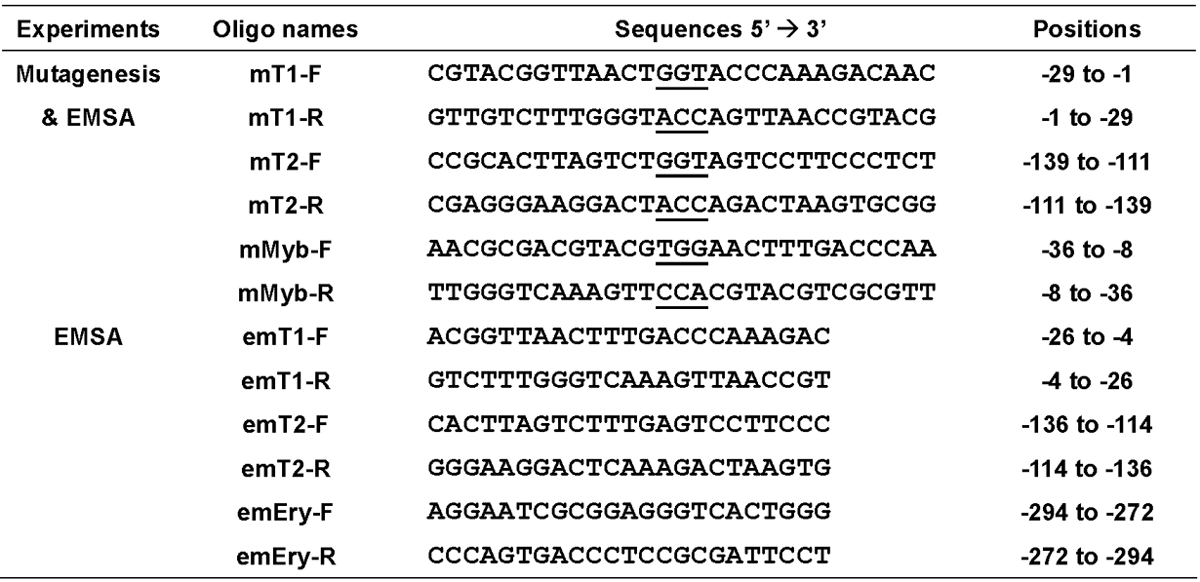


The single underlines denote mutated sequences.
